# Vertebral mobility is a valuable indicator for predicting and determining bone union in osteoporotic vertebral fractures: a conventional observation study

**DOI:** 10.1186/s13018-020-01649-y

**Published:** 2020-05-05

**Authors:** Kozo Sato, Yuichiro Yamada, Masakazu Kogawa, Takuya Sekiguchi

**Affiliations:** 1 Department of Orthopaedic Surgery, Matsuda Hospital, 17-1 Sanezawa aza Tatsutayashiki, Izumi ku, Sendai, Miyagi 981-3217 Japan; 2 Centre for Orthopaedics and Trauma Research, Discipline of Orthopaedics and Trauma, University of Adelaide, Adelaide, SA 5005 Australia; 3 Department of Orthopaedic Science, Iwate Prefectural Central Hospital, 1-4-1, Ueda, Morioka, Iwate, 020-0066 Japan

**Keywords:** Osteoporosis, Vertebral fracture, Vertebral mobility, Vertebral deformity, Bone union, Conservative treatment

## Abstract

**Background:**

Conservative treatments for osteoporotic vertebral fractures (OVFs) have not been standardized, and criteria for determining bone union have not been established. To determine bone union, we have adopted a cutoff value of 1.0 mm for vertebral mobility (V-mobility), defined as the difference in anterior vertebral height (Ha) between lateral radiographs taken in weight-bearing and non-weight-bearing positions. The present study aimed to investigate the usefulness of V-mobility for determining bone union and predicting bone union at 6 months after OVF onset.

**Methods:**

The study included 54 acute OVFs from T11 to L3 in 53 patients (12 males, 41 females; mean age 82 years; age range 55–97 years) who were hospitalized at ≤ 3 weeks after OVF onset. Vertebral deformity (V-deformity) and V-mobility were evaluated in accordance with Ha on lateral radiographs taken in the sitting position (SIT), lateral decubitus position (DEC), and supine position (SUP). OVFs showing V-mobility of ≤ 1.0 mm between SIT and DEC radiographs and no intravertebral cleft on DEC radiograph were defined as semi-union, while those showing V-mobility of ≤ 1.0 mm between SIT and SUP radiographs and no intravertebral cleft on SUP radiograph were defined as bone union. We calculated the bone union rates including semi-unions associated with V-mobility cutoff values of 1.0 mm, 1.5 mm, and 2.0 mm and estimated cutoff values for V-mobility at 5 weeks after OVF onset to predict bone union at 6 months after OVF onset.

**Results:**

The cumulative number of bone unions including semi-unions was more influenced by the different V-mobility cutoff values in Ha for determining bone union in the earlier period compared with the later period in the time course of OVF. Receiver-operating characteristic curve analyses revealed that V-mobility cutoff value of 2.1 mm in Ha between SIT and DEC radiographs at 5 weeks after OVF had moderate accuracy for predicting bone union including semi-union at 6 months after OVF. The mean V-deformity value on SIT radiographs did not progress significantly.

**Conclusion:**

V-mobility in the early stage after OVF can predict bone union at 6 months after OVF and is a useful quantitative indicator for determining bone union.

## Background

The primary treatment modalities for osteoporotic vertebral fractures (OVFs) are conservative, including analgesia, bed rest, and physiotherapy [1]. However, conservative treatments can lead to non-union or pseudoarthrosis of OVFs in some patients [2] and result in a delayed neurological deficit that requires surgical intervention [3–9]. Such delayed onset of a neurological deficit suggests that the initial OVF evaluation and consequent follow-up during the early stages may have been inappropriate. A study on the causes of delayed neurological deficits after delayed vertebral collapse concluded that the most important preventative factors were correct initial diagnosis, fixation, and rest [10].

The initial evaluation of a suspected OVF includes assessments by plain radiography and magnetic resonance imaging. Vertebral mobility (V-mobility) is defined as the difference in vertebral height on radiographs taken with the patient in a weight-bearing (sitting) position versus a non-weight-bearing (lateral decubitus or supine) position. V-mobility was reported to be useful for OVF evaluation during treatment and was employed to diagnose acute OVF [11, 12], detect occurrence of bone union [13, 14] or delayed union [15–21], and predict delayed union [22].

In 2008, Kawasaki et al. [12] reported a V-mobility cutoff value of 2.0 mm in anterior vertebral height (Ha) for diagnosing fresh OVF. V-mobility of 2.0 mm in Ha is visually recognizable on radiographs without measurements. Thus, since 2008, we have adopted V-mobility of 1.0 mm in Ha between radiographs in the sitting position (SIT) and supine position (SUP) as the cutoff value for determining bone union following the digitization of radiography. In 2014, Niimi et al. [11] reported the same cutoff value for diagnosing acute OVF as Kawasaki et al. [12].

Chen et al. [16] reported a V-mobility cutoff value in Ha for non-mobile OVF in a study on mobile intravertebral clefts in OVFs in 2011, while Kitaguchi et al. [23] reported that bony union could be defined as the absence of a vertebral cleft or abnormal motion in 2019. The cutoff value for V-mobility in both of these reports was 2.0 mm in Ha. However, a cutoff value for bone union in OVF, which would be essential for standardization of OVF treatment, has not been established as described later in the text.

The present study aimed to report the results of conservative treatments based on our predetermined protocol with V-mobility cutoff value of 1.0 mm for determining bone union, and to calculate the bone union rates based on V-mobility cutoff values of 1.5 mm and 2.0 mm to demonstrate the differences in bone union rates determined by these cutoff values compared with the current cutoff value of 1.0 mm. We also aimed to calculate cutoff values of V-mobility in Ha at 5 weeks after OVF onset for predicting bone union estimated with cutoff values of 1.0 mm, 1.5 mm, and 2.0 mm for bone union at 6 months after OVF.

## Methods

### Patient selection

A total of 118 patients with primary OVFs were hospitalized in a convalescent rehabilitation ward because their pain was too severe for self-management at home in the period from October 2009 to August 2016. OVF was diagnosed on plain radiographs and magnetic resonance images. The inclusion criteria were primary OVFs of wedge and flat vertebrae from T11 to L3, acute OVFs within 3 weeks from OVF onset, and OVFs with three available radiological evaluations. The exclusion criteria were pathological fractures (tumor or infection) and steroid-induced osteoporosis. The following OVFs were also excluded: concave vertebrae without V-mobility in Ha because of intact vertebral cortices, postsurgical OVFs, current OVFs in patients treated elsewhere as inpatients, and OVFs with low-quality radiographs.

Finally, 53 eligible patients with 54 OVFs were enrolled in this conventional observation study and provided written informed consent. The patients comprised 12 males and 41 females, with a mean age of 82 years (range 55–97 years). There were five OVFs at T11, 16 at T12, 20 at L1, seven at L2, and six at L3.

### Management of patients

Most patients were rested in bed until the severity of their back pain subsided sufficiently to enable them to adopt SIT, while those with middle-column injuries were rested in bed for 3 weeks regardless of pain status. As we had previously recognized that the difference in vertebral deformity (V-deformity) represented by Ha in SIT versus lateral decubitus position (DEC) was smaller than that in SIT versus SUP, we asked the patients to lie on their bed in DEC to prevent loosening of the fracture site in SUP until detection of bone union after discharge. We confirmed similar difference in V-deformity between SIT versus DEC and SIT versus SUP in the present study as described later in the text. A U-shaped walking aid was used to lessen the compressive and forward-bending forces on the affected vertebrae. Elastic corsets were usually applied, while semi-hard corsets were used for patients with middle-column injuries. Regular exercise involving walking and trunk muscle strengthening was implemented.

### Radiological assessment

**Fig. 1:**
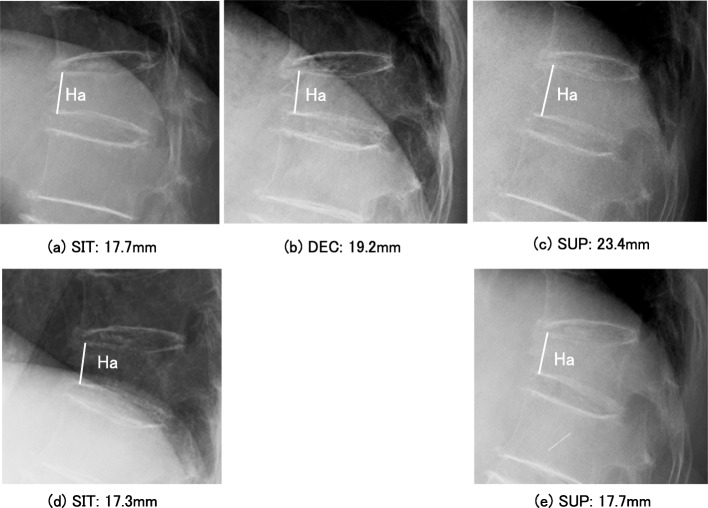
Radiological evaluation of OVFs. V-deformity in OVF was evaluated by reference to Ha on lateral radiographs taken in SIT, DEC, and SUP. **a**, **b**, and **c** show lateral radiographs of a T12 OVF in SIT, DEC, and SUP, respectively, in the initial evaluation at 12 days after OVF onset, and **d** and **e** show radiographs taken in SIT and SUP, respectively, in the final evaluation at 3 months after OVF onset in an 81-year-old female. The difference in Ha between **a**, **b**, and **c** was > 1.0 mm and that between **d** and **e** was < 1.0 mm, demonstrating no V-mobility. OVF, osteoporotic vertebral fracture; V-deformity, vertebral deformity; Ha, anterior vertebral height; SIT, sitting position; DEC, lateral decubitus position; SUP, supine position; T12, 12th thoracic vertebra; V-mobility, vertebral mobility

Plain lateral radiographs were taken in SIT, DEC, and SUP, with a tube-to-film distance of 120 cm. The images were obtained with a RadiForce MX215 (Eizo Co. Ltd., Tokyo, Japan) and had a resolution of 1200 × 1600 pixels. The radiological measurements included the dimensions of the vertebral bodies (Ha [millimeter] and posterior vertebral height [Hp, millimeter]), V-mobility defined as change in Ha between SIT and DEC radiographs or between SIT and SUP radiographs (Fig. 1), and local kyphosis angle (LKA, degrees) defined as Cobb’s angle between the cranial endplate of the vertebra cranial to the affected vertebra and the caudal endplate of the vertebra caudal to the affected vertebra.

Precision errors were calculated for Ha and Hp on radiographs of five randomly selected fractured and intact vertebrae and expressed as percentage coefficients of variation. The respective coefficients of variation for affected and intact vertebrae were 2.0% ± 1.02% and 1.1% ± 0.7% for Ha and 1.5% ± 0.4% and 0.9% ± 0.5% for Hp.

Reliability of measurements was assessed as follows. The Ha and Hp of the affected vertebrae, Hp of the cranial and caudal vertebrae, and LKA were independently measured by two authors (KS and YY). The intraobserver and interobserver reliability of the measurements was assessed by intraclass correlation coefficients based on 45 measurements of five dimensions on SIT, DEC, and SUP radiographs of three patients with OVFs. The intraobserver reliability was 0.989 (*p* < 0.001) and 0.996 (*p* < 0.001), respectively, while the interobserver reliability was 0.985 (*p* < 0.001).

### Definitions of bone union and semi-union

Bone union was defined as an affected vertebra with V-mobility of ≤ 1.0 mm between SIT and SUP radiographs and no intravertebral cleft on SUP radiograph, while semi-union was defined as an affected vertebra with V-mobility of ≤ 1.0 mm between SIT and DEC radiographs and no intravertebral cleft on DEC radiograph.

### Radiological follow-up

The initial evaluation was principally based on SIT and SUP radiographs to demonstrate the maximal V-mobility. Subsequent radiographs were taken every 3 weeks during hospitalization and every 6 weeks at the outpatient clinic in SIT and DEC to prevent loosening of the fracture site in SUP. After detection of semi-united or similarly stable OVFs on SIT and DEC radiographs, patients were assessed by SIT and SUP radiographs to ascertain OVF union. When bone union was detected, follow-up for a current OVF was finished, and the measurement at this time was defined as the final evaluation. When a semi-union was not followed-up, the semi-union was treated like a bone union, and the measurement at this time was defined as the final evaluation. The reason why a semi-union was treated like a bone union is described later in the text. Accordingly, the period from OVF onset to final evaluation varied among individual patients.

The number of OVFs available for predicting bone union at 6 months after OVF by receiver-operating characteristic (ROC) curve analysis on SIT and DEC radiographs was 26 OVFs at the initial evaluation and 44 OVFs at 5 weeks after OVF.

### Statistical analysis

Comparisons between two groups were made by a Student’s *t*-test. The correlation between V-deformity and LKA was calculated by single regression analysis. Comparisons of V-deformity and LKA in the three radiographic positions were made by one-way ANOVA followed by Bonferroni post hoc test. Cutoff values for predicting bone union at 6 months after OVF were calculated by ROC curve analysis. Data were presented as mean ± SD. Statistical test results were considered significant at *p* < 0.05. All *p* values were two-sided. Analyses were performed using BellCurve for Excel (Social Information Service, Tokyo, Japan).

## Results

### V-deformity presented as Ha and LKA

The extent of V-deformity presented as Ha and LKA differed significantly among SIT, DEC, and SUP radiographs by one way ANOVA (*p* < 0.001) (Table 1). The significant differences between each group was confirmed by Boneferroni test.

**Table 1:** Extent of V-deformity presented as anterior vertebral height and local kyphosis angle in SIT, DEC, and SUP

Dimension	Sitting position	Lateral decubitus position	Supine position
Anterior vertebral height (mm)	18.3 ± 3.3	20.7 ± 2.8*	24.3 ±3.5**^¶¶^
Local kyphosis angle (degrees)	26.2 ± 12.6	19.8 ± 12.5**	15.4 ± 10.3**^¶^

The V-deformity and local kyphosis angle were measured on radiographs in 15 patients with OVFs without middle-column injury at the initial evaluation. Because radiographs taken in SIT are usually different from those in DEC or SUP according to enlargement of the radiographs, measurements of Ha on SIT radiographs were adjusted by comparing the Ha of the adjacent vertebra on radiographs in SIT with those in DEC or SUP

*OVF* osteoporotic vertebral fracture, *V-deformity* vertebral deformity, *Ha* anterior vertebral height, *SIT* sitting position, *DEC* lateral decubitus position, *SUP* supine position

Boneferroni test *p < 0.01, **p < 0.001, SIT vs. DEC or SUP

^¶^*p* < 0.01, ^¶¶^*p* < 0.001, DEC vs. SUP

### Time course of bone healing and treatment outcomes in OVFs

The mean duration from OVF onset to hospitalization and follow-up was 6.7 ± 6.5 days and 216.3 ± 238.9 days, respectively. The initial evaluation included 25 OVFs in SIT and SUP and 29 OVFs in SIT and DEC. The final evaluation comprised 34 OVFs in SIT and SUP and 13 OVFs in SIT and DEC, excluding seven drop-out OVFs.

Regarding the time course of bone healing, the numbers of bone unions and semi-unions that were finally evaluated at each month together with the numbers of drop-out OVFs are shown in Table 2. About two-thirds of the OVFs were united or semi-united within 6 months after OVF onset, and the remaining OVFs were united thereafter. The duration from OVF onset to hospitalization in the latter OVFs was significantly longer than that in the former OVFs (10.7 ± 6.3 days vs. 4.4 ± 5.6 days, *p* = 0.001).

**Table 2:** Time course of bone healing of OVFs at each month after OVF onset

	Time after OVF onset (months)							
	3	4	5	6	9	12	>12	Total
Bone union	4	10	4	1	5	6	4	34
Semi-union^#^	5	4	1	0	0	0	2	12
Non-union	0	0	0	0	0	0	1	1
Drop-out	3	1	0	0	3	0	0	7

^#^Semi-union in the final evaluation, as described in detail in the “Radiological follow-up” section

The characteristics and final outcomes of the treatments for OVFs are shown in Table 3. In the 47 OVFs after excluding the seven drop-out OVFs, the cumulative rates of bone union and semi-union were 72.4% and 25.5%, comprising 97.9% in the final evaluation.

**Table 3:** Characteristics and outcomes of treatments for OVFs

Characteristics and treatment outcomes	46 patients, 47 OVFs
Age (years)	82 (67–97)
Sex (male/female)	9/37
BMI	21.8 ± 4.0
Duration from onset of OVF to hospitalization (days)	6.9 ± 6.6 (0–20)
Duration from onset of OVF to initial evaluation (days)	13.3 ± 10.6 (0–37)
Type of OVF (wedge/flat)	42/5
Number of prevalent OVFs	2.7 ± 1.6 (1–7)
Duration of hospitalization (days)	75.6 ± 23.6
Duration of following-up (days)	231.1 ± 252.4
Anterior vertebral mobility (mm)	
Initial evaluation	4.7 ± 2.8
Final evaluation	0.04 ± 0.6
Comparison between initial and final evaluations	*p* < 0.001^¶^
Anterior vertebral height (mm)^#^	
Initial evaluation	18.2 ± 4.3
Final evaluation	18.0 ± 5.1
Comparison between initial and final evaluations	*p* = 0.64^¶^
Local kyphosis angle (degrees)^#L^	
Initial evaluation	23.6 ± 12.6
Final evaluation	26.5 ± 14.2
Comparison between initial and final evaluations	*p* = 0.007^¶^
Bone union	34 (72.4%)
Semi-union	12 (25.5%)
Non-united OVF	1 (2.1%)

*OVF* osteoporotic vertebral fracture, *wedge-type OVF* OVF with reduced anterior vertebral height, *flat-type OVF* OVF with reduced anterior, middle, and posterior vertebral heights by > 20% of the average values for the adjacent cranial and caudal vertebrae [24]

^#^These dimensions were measured on radiographs taken in the sitting position

^L^Local kyphosis angle was measured in 44 OVFs after excluding radiographs with low quality

^¶^Paired Student’s *t*-test

The mean V-deformity value on SIT radiographs did not differ significantly between the initial and final evaluations (Fig. 1, Table 3). The LKA showed slight but significant progression (*p* = 0.007), related to the change in V-deformity (*n* = 44, *r* = 0.40, *p* = 0.005). One OVF showed V-mobility of < 1.0 mm in Ha but had a linear intravertebral cleft on DEC radiograph that remained at 2 years after OVF onset, and was therefore classified as non-union according to the Standard for Evaluation of Vertebral Fracture [24]. There were no neurological deficits.

### Cumulative numbers of bone unions and semi-unions according to the three kinds of V-mobility in Ha

**Fig. 2:**
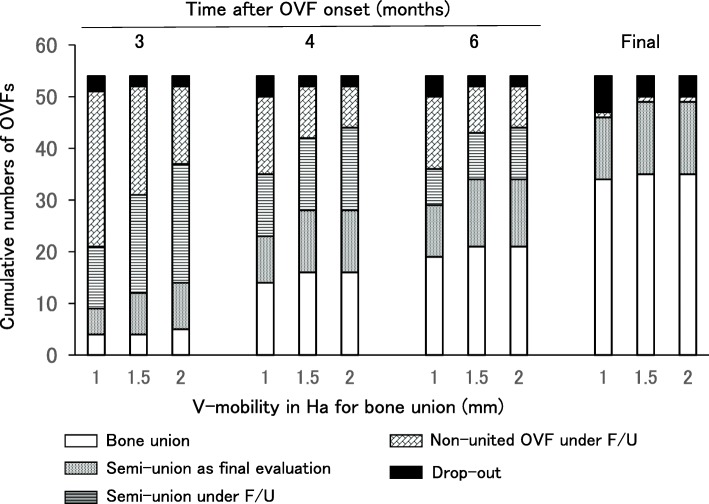
Cumulative numbers of bone unions and semi-unions of OVFs. The cumulative numbers at 3, 4, and 6 months after OVF onset and in the final evaluation according to V-mobility cutoff values of 1.0 mm, 1.5 mm, and 2.0 mm of Ha for bone union are shown. The semi-unions except for those determined to be semi-united as the final evaluation (semi-union as final evaluation) were under follow-up until the detection of bone union (semi-union under F/U). When the V-mobility in Ha for bone union was set at 1.5 mm and 2.0 mm, there was no additional bone union and the one additional OVF, respectively, at 3 months after OVF. At 4 months after OVF, there were two additional OVFs at both cutoff values. When the V-mobility in Ha for semi-union was set at 1.5 mm and 2.0 mm, the additional number of semi-unions seemed to increase more clearly than that of bone unions, particularly at 3 months after OVF. OVF, osteoporotic vertebral fracture; V-mobility, vertebral mobility; Ha, anterior vertebral height; F/U, follow-up; Final, final evaluation

The cumulative numbers of bone unions and semi-unions in OVFs at 3, 4, and 6 months and in the final evaluation are shown in Fig. 2. The OVFs under follow-up gradually progressed to semi-union and then to bone union, while the semi-unions under follow-up progressed to bone union or remained as semi-union in the final evaluation. Besides the cumulative numbers of bone unions and semi-unions determined by the V-mobility cutoff value of 1.0 mm in Ha, the numbers were calculated for V-mobility cutoff values of 1.5 mm and 2.0 mm in Ha. The cumulative rates of bone unions and semi-unions including semi-unions under follow-up at 3 months after OVF were 41.2% (21 OVFs), 59.6% (31 OVFs), and 71.2% (37 OVFs), respectively, for V-mobility cutoff values of 1.0 mm, 1.5 mm, and 2.0 mm of Ha for bone union. The corresponding rates at 4 months after OVF were 70.0% (35 OVFs), 80.8% (42 OVFs), and 84.6% (44 OVFs), respectively. The differences between the respective union rates became smaller over time, and the final union rates were 97.9% (46 OVFs), 98.0% (49 OVFs), and 98.0% (49 OVFs), respectively. In 90% of cases in this study, bone union arose from a semi-union situation.

### V-mobility cutoff value in Ha for predicting bone union at 6 months after OVF

ROC curves were used to investigate the relationships between V-mobility in Ha on SIT and DEC radiographs at the first two evaluations and bone union including semi-union at 6 months after OVF.

Two OVFs were not evaluated at 6 months after OVF onset, and four OVFs had dropped out by 6 months after OVF onset. Twenty-two OVFs at the initial evaluation and two OVFs at the second evaluation with an interval of about 3 weeks after the initial evaluation were evaluated on SIT and SUP radiographs. Two OVFs at the second evaluation that were evaluated at > 6 weeks after the initial evaluation were excluded from the analysis. Accordingly, 26 OVFs at the initial evaluation and 44 OVFs at the second evaluation were available for the ROC curve analysis.

In the 26 OVFs, the initial evaluation was conducted at 2–3 weeks (18.2 ± 10.0 days) after OVF onset. In the 44 OVFs, the initial evaluation was conducted at about 2 weeks (12.5 ± 10.2 days) after OVF onset, and the second evaluation was conducted at about 5 weeks (34.9 ± 10.2 days) after OVF onset with an interval of 3 weeks from the initial evaluation.

**Fig. 3:**
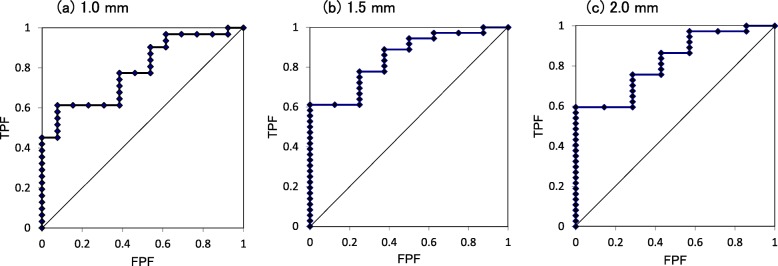
ROC curves at 5 weeks after OVF. ROC curves were used to investigate the relationships between anterior vertebral mobility and bone union (including semi-union) at 6 months after OVF. Bone union was determined by cutoff values for anterior vertebral mobility of 1.0 mm (**a**), 1.5 mm (**b**), and 2.0 mm (**c**). The distance from the top left corner of the ROC curve was used to estimate the cutoff values for predicting bone union at 6 months after OVF, which are shown in Table 4. ROC, receiver-operating characteristic; OVF, osteoporotic vertebral fracture; TPF, true-positive fraction; FPF, false-positive fraction

For Ha, the cutoff values for V-mobility at 5 weeks after OVF for predicting bone union at 6 months after OVF were 2.1 mm, 3.0 mm, and 3.0 mm, respectively, using V-mobility cutoff values for determining bone union of 1.0 mm, 1.5 mm, and 2.0 mm, with a moderate degree of predictability accuracy (Fig. 3, Table 4). The cutoff values for V-mobility at the initial evaluation showed low accuracy for predicting bone union at 6 months after OVF.

**Table 4:** Cutoff values for V-mobility at 5 weeks for predicting bone union at 6 months after OVF

	V-mobility cutoff values in Ha for determining bone union		
	1.0 mm	1.5 mm	2.0 mm
V-mobility cutoff values in Ha for predicting bone union (mm)	2.1	3.0	3.0
AUC	0.787	0.847	0.822
FPF	0.077	0.250	0.286
TPF	0.613	0.778	0.757
Bone union/non-united OVFs at 6 months after OVF onset	31/13	36/8	37/7
Percentage of bone union	70.5	81.8	84.1
Number of OVFs for ROC curve analysis on SIT and DEC radiographs	44	44	44

Cutoff values for V-mobility in anterior vertebral height at 5 weeks after OVF onset for predicting bone union at 6 months after OVF onset were determined by ROC curves (bone union includes semi-union)

*OVF* osteoporotic vertebral fracture, *ROC* receiver-operating characteristic, *Ha* anterior vertebral height, *V-mobility* vertebral mobility, *AUC* area under the curve, *FPF* false-positive fraction, *TPF* true-positive fraction, *SIT* sitting position, *DEC* lateral decubitus position

## Discussion

In the present study, the cumulative numbers of bone unions including semi-unions were variable, particularly in the early months after OVF, depending on the V-mobility cutoff value for bone union. The cumulative number of bone unions was less apparently influenced by the three V-mobility cutoff values for bone union, 1.0 mm, 1.5 mm, and 2.0 mm, than the cumulative number of semi-unions.

The ROC curve analysis indicated that V-mobility cutoff values on SIT and DEC radiographs taken at 5 weeks after OVF could predict bone union including semi-union at 6 months after OVF with moderate accuracy. The V-mobility cutoff values obtained in the initial evaluation showed lower accuracy.

The one-third of OVF patients hospitalized at more than 10 days on average after OVF onset required a longer period for bone union than the remaining patients who were hospitalized at 4 days on average.

It was reported that earlier adequate intervention is a key factor for OVF treatment [10, 13], and it was found that even 1 week earlier hospitalization leads to significantly earlier bone healing [25].

The reason why the cumulative number of bone unions was less influenced by the three V-mobility cutoff values for bone union is likely to be that the bone unions were determined after they had become stable with V-mobility of less than about 1.0 mm in Ha on SIT and DEC radiographs. A semi-union was treated like a bone union, because a semi-union was already stable at ≤ 1.0 mm of V-mobility in Ha on SIT and DEC radiographs, and it was confirmed in this study that 90% of bone unions arose from a semi-union situation.

Previous reports describing criteria for bone union in OVF are very limited. In one report, bone union was determined when the vertebral body did not show any changes in shape between the standing and supine positions without measurements of vertebral dimensions [13]. In another report, vertebrae were referred to as “settled” when dynamic mobility was not observed between SIT and SUP radiographs [14]. In such cases, the actual V-mobility value remained unclear because V-mobility was not measured or the V-mobility value was not described, and therefore the judgement for no V-mobility without measurements requires prior confirmation of reliability.

Because an intravertebral cleft was reported to occur at about 3 weeks after OVF onset with inadequate treatment during the early stage after OVF [10] and to be one of the findings indicating delayed union [20, 22] or non-union [2, 15, 17, 19, 26], lack of a cleft should be included as a criterion for determining bone union.

Therefore, bone union should be determined based on a certain extent of V-mobility without an intravertebral cleft, and the extent to which V-mobility for bone union is clinically useful requires clarification. Kitaguchi et al. [23] reported effects of weekly teriparatide administration for bone union in patients with OVFs at 8 and 12 weeks after OVF onset based on a cutoff value of 2.0 mm in Ha for determining bone union. If V-mobility of 2.0 mm in Ha is a cutoff value for bone union, it is preferable to be confirmed whether OVFs determined to be united based on this V-mobility cutoff value in Ha have settled without developing an intravertebral cleft or progression of V-deformity thereafter. As a V-mobility cutoff value for bone union has not been established, V-mobility of about 1.0 mm in Ha may be a preferable cutoff value for bone union, because this V-mobility value can be considered the minimal measurable value on radiographs.

Takahashi et al. [22] reported a ROC curve analysis indicating that vertebral angular motion of ≥ 5° on SIT and SUP radiographs at both enrollment and the 1-month follow-up was a risk factor for delayed union at 6 months after OVF, with low accuracy at enrollment. V-mobility cutoff values at about 5 weeks after OVF may be more accurate for predicting bone union than those in the early stage after OVF.

The present results suggest that it may be very important to treat patients with OVFs in an adequate manner, including hospitalization at an early stage after OVF based on evaluation of OVF severity by reference to pain, V-deformity, and V-motility, and to manage OVF patients by reference to the V-mobility cutoff value at 5 weeks after OVF onset, as a promising indicator for favorable outcomes at 6 months after OVF onset.

## Study limitations

First, only a very small number of OVFs were included, resulting in limited statistical power. Second, the case registration period was long at up to 7 years. However, the characteristics of the patients, including age, sex, BMI, period from OVF onset to hospitalization, hospitalization days, and number of prevalent OVFs, did not differ significantly between cases before and after 2013 as the middle of the registration period. Third, follow-up radiographs were taken in SIT and DEC while avoiding SUP. Thus, radiographs in SIT and SUP were not obtained for some patients because their OVFs were stable in SIT and DEC, and these OVFs were treated as semi-unions. Therefore, it is likely that V-mobility was underestimated and the bone union rate was overestimated on SIT and DEC radiographs, particularly during the early stage after OVF. Fourth, there was no control group of patients who lay in SUP during treatment, and therefore the potential advantage of lying in DEC for bone union and/or prevention of V-deformity remains unclarified.

## Conclusions

The ROC curve analysis revealed that V-mobility of ≤ 2.1 mm in Ha on SIT and DEC radiographs at 5 weeks after OVF may be a promising indicator for obtaining bone union including semi-union at 6 months after OVF. The difference in bone union rates using V-mobility cutoff values of 1.0 mm, 1.5 mm, and 2.0 mm in Ha for bone union, particularly during the initial few months after OVF, indicated the necessity of determining a cutoff value for bone union, which will enable comparisons with other results and standardization of OVF treatment.

## Data Availability

The datasets used and/or analyzed during the current study are available from the corresponding author on reasonable request.

## Abbreviations

AUC: Area under the curve
DEC: Lateral decubitus position
FPF: False-positive fraction
Ha: Anterior vertebral height
Hp: Posterior vertebral height
LKA: Local kyphosis angle
OVF: Osteoporotic vertebral fracture
ROC: Receiver-operating characteristic
SIT: Sitting position
SUP: Supine position
TPF: True-positive fraction
V-deformity: Vertebral deformity
V-mobility: Vertebral mobility
